# Adversarially Training MCMC with Non-Volume-Preserving Flows

**DOI:** 10.3390/e24030415

**Published:** 2022-03-16

**Authors:** Shaofan Liu, Shiliang Sun

**Affiliations:** School of Computer Science and Technology, East China Normal University, Shanghai 200062, China; 51194506021@stu.ecnu.edu.cn

**Keywords:** Hamiltonian Monte Carlo, flow models, Markov chain Monte Carlo, statistical pattern recognition, Bayesian machine learning

## Abstract

Recently, flow models parameterized by neural networks have been used to design efficient Markov chain Monte Carlo (MCMC) transition kernels. However, inefficient utilization of gradient information of the target distribution or the use of volume-preserving flows limits their performance in sampling from multi-modal target distributions. In this paper, we treat the training procedure of the parameterized transition kernels in a different manner and exploit a novel scheme to train MCMC transition kernels. We divide the training process of transition kernels into the exploration stage and training stage, which can make full use of the gradient information of the target distribution and the expressive power of deep neural networks. The transition kernels are constructed with non-volume-preserving flows and trained in an adversarial form. The proposed method achieves significant improvement in effective sample size and mixes quickly to the target distribution. Empirical results validate that the proposed method is able to achieve low autocorrelation of samples and fast convergence rates, and outperforms other state-of-the-art parameterized transition kernels in varieties of challenging analytically described distributions and real world datasets.

## 1. Introduction

Markov chain Monte Carlo (MCMC) is one of the most powerful approaches to sample from complex target distributions in statistical pattern recognition and Bayesian machine learning. It has been widely employed in probabilistic modeling and inference [1,2]. MCMC methods approximate target distributions by generating samples from a proposal distribution depending on the last sample, and ensure that the samples converge to the target distribution by satisfying the detailed balance [3]. In theory, we can arbitrarily choose a proposal distribution and employ the Metropolis–Hastings (MH) [4] algorithm to satisfy the detailed balance. However, the specific choice of the proposal distribution has a significant effect on the convergence and mixing speed [5,6]. For instance, a Gaussian distribution centered on the current state is a regular choice for the proposal distribution, which is also called a random walk proposal [7,8]. Although it has a particularly nice intuition, the proposal scales poorly with the increase of dimensions and complexity of the target distribution [9,10].

Hamiltonian Monte Carlo (HMC) [7] introduces auxiliary momentum variables *v* to extend the state space to (x, v). In every update step of HMC, the leapfrog discretization scheme is used to update *x* and *v* alternately [11]. Before a new update step, the auxiliary variables are resampled to explore in the new equal-energy surface (or iso-probability contour) so that *x* could change greatly in a systematic way. As a result, HMC can traverse a long distance in the state space with a single MH step and prevent the random walk behavior [7,12]. However, HMC can suffer dramatically in highly complex, multi-modal distributions, for it is difficult to traverse low-density regions [13,14].

The core problem of MCMC is to build transition kernels (also known as the proposal distribution) that can explore and sample from the target distribution efficiently. Recently, training MCMC transition kernels parameterized by deep neural networks achieves great success [13,15,16]. A-NICE-MC [15] uses volume-preserving flows [17] as transition kernels and trains via generative adversarial network (GAN) [18,19]. L2HMC [13] uses non-volume-preserving flows incorporating gradient information of the target distribution as transition kernels, and the sampler is trained to maximize the expected squared jumped distance which is equivalent to minimizing the lag-one autocorrelation [20].

A-NICE-MC uses volume-preserving flows as the transition kernels bring the random walk behavior when traversing between different energy levels, and then they can get correct samples through one MH step. They maintain a buffer to save the sample points, use samples from the buffer as the learning objective of their transition kernels, and then the buffer is updated with the new sample points [15]. In this way, they do not need to compute the gradient of the target distribution since the sample points in the buffer already contain the geometric information of the target distribution. However, although eliminating the calculation of the gradient reduce the computational burden, without the guidance of gradient information, this exploration is almost random. L2HMC introduces neural networks into the leapfrog integrator to construct more flexible transition kernels, where the gradient information of the target distribution is used as the input of neural networks. Consequently, when exploring the same region twice, the gradient information of the region also needs to be calculated twice since we can not record all gradients of the target distribution. Thus the gradient information can not be exploited efficiently in the training process, which may result in unnecessarily long training time.

To solve these issues, we design a new method that uses non-volume-preserving flows as transition kernels to mix faster for the target distribution. Specifically, we divide the training process of the parameterized transition kernels into the exploration stage and training stage. In the exploration stage, we use a gradient-based transition kernel to explore the target distribution as much as possible, and we can design powerful exploration operators without the restriction of convergence. We save samples of the exploration stage and use these samples to record the geometry of the target distribution. In the adversarial training stage, we optimize the parameters of the transition kernels by minimizing the distance between samples generated from transition kernels and samples from the exploration stage. Then samples generated from the trained transition kernels are accepted through MH steps [21]. The accepted samples will be used to replace some of the samples collected in the previous exploration stage. With the increase of the training iterations, we can generate samples with better quality at a low computation cost since the collected samples record the geometry of the target distribution. As a result, in the training stage, we need not compute the gradient to get a high acceptance rate like L2HMC or vanilla HMC. Gradients are only needed in the exploration stage.

The rest of this paper is organized as follows. In Section 2, we firstly introduce the necessary background on MCMC methods and non-volume-preserving flows. We present the proposed method in detail in Section 3. In Section 4, we describe the core ideas of A-NICE-MC and L2HMC, we also introduce the motivation of our method in this section. Experiments are given in Section 5. Finally, we conclude this paper in Section 6.

## 2. Background

### 2.1. Markov Chain Monte Carlo and Metropolis–Hasting Algorithm

MCMC methods [2] aim to construct an ergodic Markov chain converging to p(x) under a target density $p\left(x\right)=\frac{\tilde{p}\left(x\right)}{Z_{p}}$, where $\tilde{p}\left(x\right)$ can be readily evaluated and $Z_{p}$ is an unknown constant. At each step of the algorithm, the new sample $x^{\prime }$ is obtained from the transition kernel (or proposal distribution) $K_{\theta }\left(x^{\prime }|x\right)$ which depends on the current state *x*. The MH step is utilized to make the Markov chain satisfy the detailed balance which can be written as:

(1) $$p\left(x^{\prime }\right)K_{\theta }\left(x|x^{\prime }\right)=p\left(x\right)K_{\theta }\left(x^{\prime }|x\right).$$

Specifically, a new sample $x^{\prime }$ generated from a proposal distribution $q_{\theta }\left(x^{\prime }|x\right)$ is accepted with probability $A_{\theta }\left(x^{\prime }|x\right)$ which takes the form as:

(2) $$A_{\theta }\left(x^{\prime }|x\right)=\min\left(1,\frac{p\left(x^{\prime }\right)q_{\theta }\left(x|x^{\prime }\right)}{p\left(x\right)q_{\theta }\left(x^{\prime }|x\right)}\right).$$

For vanilla HMC, assume that ξ is a state in the Hamiltonian dynamics. The transition from ξ to $\xi^{★}$ is deterministic, invertible and volume-preserving, which means that $q_{\theta }\left(\xi |\xi^{★}\right)=q_{\theta }\left(\xi^{★}|\xi \right)$ [15]. According to the change of variable formula which takes the form as:

(3) $$p_{X}\left(x\right)=p_{Z}\left(f\left(x\right)\right)\left|\det\left(\frac{\partial f(x)}{\partial x^{⊤}}\right)\right|,$$

the MH acceptance probability for the HMC proposal [22] can be simplified as:

(4) $$A_{\theta }\left(\xi^{★}|\xi \right)=\min\left(1,\frac{p\left(\xi^{★}\right)}{p\left(\xi \right)}\left|\frac{\partial q_{\theta }\left(\xi^{★}|\xi \right)}{\partial \xi^{⊤}}\right|\right),$$

where $\left|\frac{\partial q_{\theta }\left(\xi^{★}|\xi \right)}{\partial \xi^{⊤}}\right|=\frac{q_{\theta }\left(\xi |\xi^{\prime }\right)}{q_{\theta }\left(\xi^{\prime }|\xi \right)}=1$.

### 2.2. Hamiltonian Monte Carlo and Exploration on Total Energy Function

In Hamiltonian Monte Carlo (HMC) [7], we assume that the target distribution p(x) takes the form as:

$$p\left(x\right)=\frac{1}{Z_{U}}\exp\left[-U(x)\right],$$

where U(x) is interpreted as the potential energy of the dynamics, and $Z_{U}$ is an unknown constant. Auxiliary variables *v* can be interpreted as the momentum of the dynamics, and the kinetic energy takes the form as:

$$K\left(v\right)=\frac{1}{2}v^{⊤}v.$$

The total energy *H* is the sum of potential and kinetic energies:

H(x, v)=U(x)+K(v),

and the joint distribution can be written as:

$$p\left(x,\;v\right)=\frac{1}{Z_{H}}\exp\left[-H(x,v)\right].$$

When simulating Hamiltonian dynamics for a finite time, the value of *x* and *v* will change with the total energy conserving. In practice, *x* and *v* are updated through the leapfrog discretization, which for a single time step consists of:

(5) $$\begin{array}{cc} v & =v-\frac{\epsilon }{2}\partial_{x}U\left(x\right);\\ x^{\prime } & =x+\epsilon v;\\ v^{\prime } & =v-\frac{\epsilon }{2}\partial_{x}U\left(x^{\prime }\right), \end{array}$$

where ϵ is the step size.

In this way, *x* can change in a systematic way, which prevents the random walk behavior [23]. Samples of the joint distribution are obtained by traversing along the equal-energy surface of the extended state space. To explore other regions of the target distribution, the momentum variables *v* should be resampled from an isotropic Gaussian.

**Figure 1:**
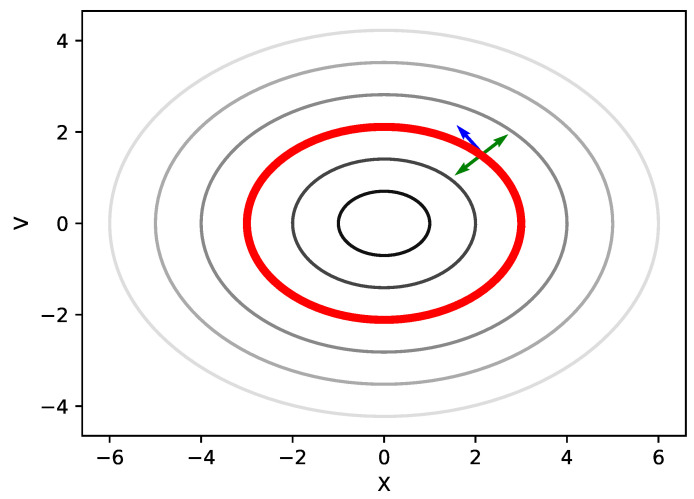
Traversing between energy levels. The blue arrow represents the deterministic exploration of an energy level. Green arrows represents the random walk between energy levels.

Consequently, as Figure 1 shows, the entire HMC can be divided into two stages: the deterministic exploration of an energy level which is represented by the blue arrow, and the random walk between energy levels which is represented by the green arrow. The contours represent different energy levels, where the energy level of the outer contour is higher [24,25]. For the volume-preserving flows, the exploration across energy levels is achieved by resampling a new momentum variable $v^{\prime }$ generated from the Gaussian distribution, and thus it can only explore regions of different energy levels in a random walk behavior which is inefficient [26,27]. Although the process of the leapfrog discretization can introduces numerical errors inevitably, the MH step can help HMC converge to the target distribution. Since HMC can well preserve the total energy, it has a high acceptance rate.

Schematic representation of deterministic sampling at a given energy level (blue arrow) and Gaussian redistribution of kinetic energy (green arrows).

If we can change the total energy greatly during exploration and guarantee that the transition kernels can converge to the target distribution, the random walk behavior of the exploration between energy levels can be prevented [28]. This idea encouraged us to develop the non-volume-preserving sampler. In our algorithm, we utilize the non-volume-preserving flows as the transition kernels, which means that $\left|\frac{\partial q_{\theta }\left(\xi^{★}|\xi \right)}{\partial \xi^{⊤}}\right|\neq 1$. Thus, to avoid a huge computational burden, the Jacobian determinant of the transition kernels should be easy to compute. Next we will introduce the non-volume-preserving flow used in this paper, which has the desired property.

### 2.3. Real-Valued Non-Volume-Preserving Flows

**Figure 2:**
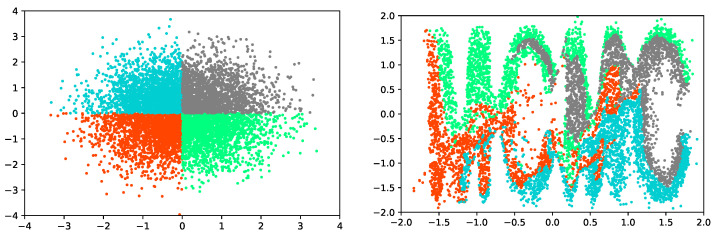
Scatter plots of normal distribution (**left**) and complex distribution of “MCMC” (**right**). We use 6 layers RNVP flows constructed by neural networks with each layer contains 512 hidden units and training with maximum likelihood estimation. The result is observed after 100,000 iterations with a learning rate of 0.0001. The batch size is set to 512.

The main idea of the real-valued non-volume-preserving (RNVP) flows [13,29,30,31] is to construct flexible and invertible architectures that enable the computation of log-likelihood on continuous data using the change of variable formula [30]. By stacking a sequence of carefully designed bijection functions which have tractable Jacobian determinant, flexible and expressible transition functions can be constructed. As illustrated in Figure 2, we use only four layers non-volumn-preserving flows to map a normal distribution to a complex distribution. This simple experiment proves the powerful expression ability of RNVP. Each bijection function of RNVP is called coupling layers with the form as:

(6) $$\begin{array}{cc} y_{1:d} & =x_{1:d}\\ y_{d+1:D} & =x_{d+1:D}⊙\exp\left[S\left(x_{1:d}\right)\right]+T\left(x_{1:d}\right), \end{array}$$

where the Jacobian of this function is:

(7) $$\frac{\partial y}{\partial x^{⊤}}=\left[\begin{array}{cc} I_{d} & 0\\ \frac{\partial y_{d+1:D}}{\partial x_{1:d}^{⊤}} & \mathrm{diag}\left(\exp\left[S\left(x_{1:d}\right)\right]\right) \end{array}\right],$$

which is triangular and where determinant can be computed simply by $\exp\left[\sum_{j}S\left(x_{1:d}\right)\right]$. *S* rescales the inputs and *T* is a translation. Arbitrarily complex transition functions can be built by stacking numerical layers. Moreover, computing the inverse of the coupling layers does not require computing the inverse of *S* and *T* [13]:

(8) $$\begin{array}{cc} x_{1:d} & =y_{1:d}\\ x_{d+1:D} & =\left[y_{d+1:D}-T\left(y_{1:d}\right)\right]⊙\exp\left[-S\left(y_{1:d}\right)\right], \end{array}$$

and its Jacobian determinant is also tractable.

Since the computation of the determinant requires $\exp\left[\sum_{j}S\left(x_{1:d}\right)\right]$ (or $\exp\left[-\sum_{j}S\left(x_{1:d}\right)\right]$ for inverse transition), we can exploit arbitrarily flexible functions such as deep neural networks as *S* and *T*. Therefore, we use RNVP to construct the transition kernels.

## 3. The Proposed Method

To overcome the problem of inefficient exploration of the volume-preserving flows, and inefficient utilization of gradient information of the target distribution, we propose NVP-MC, which exploits non-volume-preserving flows to construct transition kernels, and repeatedly utilize gradient information of the target distribution through adversarially training. In the following subsections, we will first describe the main idea of the proposed method, and then introduce how to sample from a target distribution $p_{d}$ specified by an analytic expression:

(9) $$p_{d}\left(x\right)=\frac{1}{Z}\exp\left[-U(x)\right],$$

where *Z* is an unknown normalization constant and U(x) is the potential energy function in Hamiltonian dynamics.

### 3.1. Using Non-Volume-Preserving Flows as Generator

We construct three layers of the non-volume-preserving flow model as the generator. At each layer of the generator, the update of position and momentum variables are correlative.

We firstly update auxiliary momentum variables with the following transition function:

(10) $$v^{\prime }=v+\epsilon T\left(x\right),$$

where T(x) is a translation item determined by *x*. We do not multiply a scale item to *v* like RNVP in Equation (6), because we find that it can bring much worse results, and is difficult to train. The Jacobian determinant of this transition function is I. Next we update *x* through the transition function:

(11) $$x^{\prime }=x⊙\exp\left[\epsilon S(v^{\prime })\right]+T\left(v^{\prime }\right)⊙\exp\left[\epsilon S(v^{\prime })\right],$$

where $S(v^{\prime })$ is the scale item and $T(v^{\prime })$ is the translation item that all determined by $v^{\prime }$. To simplify the calculation, we use the same item to rescale *x* and $T(v^{\prime })$, which makes the training process more stable in practice. The determinant of this transition function is $\exp\left[\epsilon S(v^{\prime })\right]$. We note that our transition kernels update *x* without dividing the *x* into two parts like other methods. When a part of *x* is updated through another part of *x*, the correlation between data will inevitably increase. Thus, we gave up this approach and only updated *x* based on *v*.

Finally, we use the same function of Equation (10) to update *v* again. The full forward update steps are shown below:

(12) $$\begin{array}{cc} v^{\prime } & =v+\frac{\epsilon }{2}T\left(x\right);\\ x^{\prime } & =x⊙\exp\left[\epsilon S(v^{\prime })\right]+T\left(v^{\prime }\right)⊙\exp\left[\epsilon S(v^{\prime })\right];\\ v^{\prime \prime } & =v^{\prime }+\frac{\epsilon }{2}T\left(x^{\prime }\right). \end{array}$$

The $\left(x^{\prime },v^{\prime \prime }\right)$ represents the next state. Then we use MH algorithm to ensure $p_{d}$ is the stationary distribution of the transition kernels. Assume that $f_{\theta }$ represents forward transition function and ξ=(x, v) represents the previous state, the MH acceptance probability takes the form as:

(13) $$A_{\theta }\left(\xi^{★},\xi \right)=\min\left(1,\frac{p\left(\xi^{★}\right)}{p\left(\xi \right)}\left|\frac{\partial f_{\theta }\left(\xi^{★}\right)}{\partial \xi^{⊤}}\right|\right),$$

where $\xi^{★}$ denotes the new state obtained from the transition kernels depending on ξ. Because the update steps of *v* and $v^{\prime }$ are volume preserving, we only need to compute Jacobian determinant of Equation (11) which is $\exp\left[\sum_{i}\epsilon S\left(v_{i}\right)\right]$, where $v_{i}$ represents the *i*-th dimension of *v*. To obtain the symmetric transition behavior and satisfy the detailed balance, we need to build the inverse transition function $f_{\theta }^{-1}$ and choose froward transition and backward transition functions with the same probability. The inverse transition function $f_{\theta }^{-1}$ takes the form as:

(14) $$\begin{array}{cc} v^{\prime } & =v^{\prime \prime }-\frac{\epsilon }{2}T\left(x^{\prime }\right);\\ x & =\left(x^{\prime }-T\left(v^{\prime }\right)⊙\exp\left[\epsilon S(v^{\prime })\right]\right)⊙\exp\left[-\epsilon S(v^{\prime })\right];\\ v & =v^{\prime }-\frac{\epsilon }{2}T\left(x\right). \end{array}$$

The Jacobian determinant of Equation (14) is $\exp\left[\sum_{i}-\epsilon S\left(v_{i}\right)\right]$ which is similar to Equation (12). The proposed method is similar to the past work of L2HMC and A-NICE-MC. Our approach incorporates adversarial training of A-NICE-MC to prevent the usage of gradient information and uses non-volume-preserving flows to construct flexible transition kernels.

### 3.2. Loss Function and Training Procedure

Now, we describe the training procedure of the proposed method. Firstly, we run a gradient-based exploration operator to explore the energy function of the target distribution and get some samples to initialize the buffer. In A-NICE-MC, HMC is chosen as the exploration operator. However, in our scheme, we do not need the exploration operator to sample exactly from the target distribution and only need to explore more regions of the target distribution.

One of the characteristic properties of high-dimensional spaces is that there is much more volume outside any given neighborhood than inside of it [9]. In other words, exploration towards the uniform distribution is inefficient in high-dimensional spaces [32]. In the *D*-dimensional spaces, the additional computational cost of evaluating a gradient compared with evaluating the function itself will typically be a fixed factor independent of *D*, whereas the *D*-dimensional gradient vector conveys *D* pieces of information compared with the one piece of information given by the function itself [23]. Therefore, we also use the gradient-based sampler as the exploration operator. Some studies have shown that the MCMC methods implemented without the detailed balance can achieve acceleration of convergence [33,34]. Thus we exploit HMC without MH steps as the exploration operator to get higher mixing performance.

At each epoch of training, we generate samples through the transition kernels and use the MH algorithm to compute acceptance probability. We treat all the samples from the transition kernels as the “fake samples”, and treat the samples from the buffer as the “true samples”. A discriminator will be trained to distinguish the “true samples” from the “fake samples”. Our transition kernel will be trained to fool the discriminator. After getting new samples, we drop some samples of the buffer in a constant ratio and insert the new accepted samples, and thereby the quality of samples in the buffer can be improved. We train our parameters in the framework of GANs, and the training objective can be formulated as:

(15) $$\begin{array}{cc} \min_{K}\max_{D}V\left(D,K\right) & =\min_{T}\max_{D}E_{x∼B(x)}\left[D\left(x\right)\right]\\ \; & -E_{z∼N(0,1)}\left[D\left(K\left(z\right)\right)\right] \end{array}$$

where *K* is our transition kernel, *D* is the discriminator network, and *B* is the buffer used to save the correct samples. To reduce the autocorrelation of samples obtained from the generator, we use the pairwise discriminator in [15]. We initialize *B* with HMC without the MH step. In fact, the samples of the uniform distribution can also be used to initialize the buffer. Because a part of samples will be dropped after every training epoch, and some new samples will be added into the buffer, the quality of samples in the buffer will improve stably during the training process. However, initial samples from the uniform distribution can not scale well in high-dimensional state spaces, it can only perform well in low-dimensional state spaces. We exactly describe our training procedure in Algorithm 1.

Unlike L2HMC, we do not exploit the gradient information of the target distribution to train the transition kernels. We have actually tried to do that, and find that the introduction of the gradient items increases the training time by tenfold but not get better results than the proposed method. Moreover, we find that L2HMC can not exploit the gradient information of the target distribution properly. We will discuss the phenomenon in detail in the next section.   
**Algorithm 1** Traing NVP-MC**Input:**
Energy function U(x), batch size *M*, learning rate α, number of iterations *N*, empty buffer *B*, transition kernels $K_{\theta }$ and $K_{\theta }^{-1}$.
1: Initialize *B* using HMC without the MH step. Initialize the parameters of the transition kernel $K_{\theta }$ and parameters of the discriminator $D_{\phi }$.

2: **for**
i=1→i=N
**do**

3:       Sample a batch ${\left\{{\left(x,v\right)}_{\left(i\right)}\right\}}_{i⩽N}$ of Gaussian noise as the start points.

4:       **for** i=1→i=M **do**

5:             Randomly sample a number *u* in open interval (0,1).

6:             Choose transition kernel: $K_{\theta }\left(x,v\right)=\left\{\begin{array}{cc} \;K_{\theta }\left(x,v\right), & 0<u<0.5\\ \;K_{\theta }^{-1}\left(x,v\right), & 1>u>0.5. \end{array}\right.$

7:             Generate the new sample $\left\{{\left(x^{\prime },v^{\prime \prime }\right)}_{\left(i\right)}\right\}$ through $K_{\theta }.$

8:             Accept the new sample with probability computed by Equation (4),

9:             and replace the samples in *B* with the accepted samples.

          **end for**

10:           Sample a batch ${\left\{{\left(x^{\prime \prime }\right)}_{\left(i\right)}\right\}}_{i⩽N}$ from *B* as the correct samples.

11:           Update the discriminator:

12:           $\phi \leftarrow \phi -\alpha \nabla_{\phi }\frac{1}{M}\sum_{i=1}^{M}\left[\log D_{\phi }\left(x_{\left(i\right)}^{\prime \prime }\right)+\log\left(1-D_{\phi }\left(K_{\theta }\left(x_{\left(i\right)}\right)\right)\right)\right].$

13:           Update the transition kernel:
14:           $\theta \leftarrow \theta -\alpha \nabla_{\theta }\frac{1}{M}\sum_{i=1}^{M}\log\left(1-D_{\phi }\left(K_{\theta }\left(x_{\left(i\right)}\right)\right)\right).$

          **end for**


## 4. Related Work

Since the choice of the proposal distribution determines the effect of the MH algorithm, many works have focused on this research. Recently, some works [13,15] exploit flexible deep neural networks or flow models [17,30] to build the proposal distribution that can mix fast for the target distribution. These algorithms outperform the vanilla Hamiltonian Monte Carlo (HMC) [35], and are the state-of-the-art methods.

### 4.1. Getting MCMC Transition Kernels through Adversarially Training

A-NICE-MC [15] aims to obtain parameterized MCMC transition kernels as the proposal distribution of MH algorithm through adversarially training. The training principle is similar to Wasserstein GANs [18]. They use a novel pairwise discriminator to reduce autocorrelation of samples by scoring two samples at a time. For every sample pair, “real data” $\left(x_{r_{1}},x_{r_{2}}\right)$ are drawn from bootstrapped samples. Assume $K_{\theta }\left(x|x^{\prime }\right)$ represents the transition kernel, the “fake data” $\left(x_{f_{1}},x_{f_{2}}\right)$ are generated by $x_{f_{1}}∼K_{\theta }\left(x|x_{f_{2}}\right)$, where $x_{f_{2}}$ is either drawn from the data distribution or generated from the transition kernel with initial noise samples. Compared with the normal discriminator, the pairwise discriminator is more sensitive among the correlation of samples. For the generation process of “fake data”, the introduction of true data makes the training procedure more stable. Inspired by HMC, A-NICE-MC also introduces auxiliary variables into the transition kernels, but without gradient information of the target distribution. They leverage volume-preserving flows as the transition kernels, which have tractable Jacobian determinants and are the same as vanilla HMC. The inputs into the transition kernels of A-NICE-MC will first be partitioned into two parts $x_{1}$ and $x_{2}$, then the transition kernels take the form as:

(16) $$\begin{array}{cc} x_{1}^{\prime } & =x_{1}\\ x_{2}^{\prime } & =x_{2}+m\left(x_{1}\right), \end{array}$$

where *m* is a neural network. Intuitively, the expression ability of the transfer kernels is not sufficient. However, the transition function can be stacked multiple layers to get an expressive model [17]. In HMC, the introduction of auxiliary variables can prevent the random walk behavior. Auxiliary variables in A-NICE-MC have a similar purpose and add randomness to the generator. In practice, they obtain exact initial samples by running HMC and use a bootstrap process [36] to generate samples.

However, A-NICE-MC suffers from the same difficulties in mixing across energy levels as HMC [13], especially when facing multi-modal distributions. For gradient-based MCMC methods, effective exploration is achieved by exploiting the differential structure of the energy function. In addition, when a region is explored twice, the gradient is also calculated twice, which is inefficient. In other words, they only exploring, but not making full use of the information of the area that has been explored.

Using gradient-based MCMC methods to get some initial samples, we can repeatedly utilize gradient information of the target distribution through adversarially training, which will bring considerable performance improvement. Therefore, we develop a new method for training flexible MCMC kernels, which not only has the powerful exploration ability of non-volume-preserving flows, but also can effectively utilize gradient information of the target distribution in an adversarially training form.

### 4.2. Parameterized Non-Volume-Preserving Transition Kernels

Vanilla HMC can be seen as an invertible volume-preserving flow, which has difficulties in mixing across energy levels [13]. Inspired by RNVP, L2HMC introduces neural networks as scale and translation items into leapfrog integrator to construct the parameterized non-volume-preserving transition kernels, which can explore the target distribution efficiently. The training objective of L2HMC aims to maximize the acceptance rate and expected squared jumped distance [20], and the loss function takes the form as:

(17) $$\ell_{\lambda }\left[\xi ,\xi^{★},A\left(\xi^{★}|\xi \right)\right]=\frac{\lambda^{2}}{\delta \left(\xi ,\xi^{★}\right)A\left(\xi^{★}|\xi \right)}-\frac{\delta \left(\xi ,\xi^{★}\right)A\left(\xi^{★}|\xi \right)}{\lambda^{2}},$$

where ξ=(x,v) and $\xi^{★}=\left(x^{\prime },\;v^{\prime }\right)$ represent the last state and the new state, respectively. λ is a scale parameter, and $\delta \left(\xi ,\xi^{★}\right)$ is the expected squared jumped distance between these two states. $A\left(\xi^{★}|\xi \right)$ denotes the acceptance probability for the new state $\xi^{★}$. Intuitively, the training objective encourages the parameterized transition kernels to get low autocorrelation of samples and meanwhile keep a high acceptance rate. Maximizing expected squared jumped distance is equivalent to minimizing the lag-one autocorrelation [20].

In L2HMC, scale and transition items are all controlled by neural networks. The parameterized transition kernels take the form:

(18) $$\begin{array}{cc} v^{\prime } & =v⊙\exp\left[\frac{\epsilon }{2}S_{v}\left(\zeta_{1}\right)\right]-\frac{\epsilon }{2}\left(\partial_{x}U\left(x\right)⊙\exp\left[\epsilon Q_{v}\left(\zeta_{1}\right)\right]+T_{v}\left(\zeta_{1}\right)\right)\\ x^{\prime } & =x_{{\bar{m}}^{t}}+m^{t}⊙\left(x⊙\exp\left[\epsilon S_{x}\left(\zeta_{2}\right)\right]+\epsilon \left(v^{\prime }⊙\exp\left[\epsilon Q_{x}\left(\zeta_{2}\right)\right]+T_{x}\left(\zeta_{2}\right)\right)\right)\\ x^{\prime \prime } & =x_{m^{t}}^{\prime }+{\bar{m}}^{t}⊙\left[x^{\prime }⊙\exp\left[\epsilon S_{x}\left(\zeta_{3}\right)\right]+\epsilon \left(v^{\prime }⊙\exp\left[\epsilon Q_{x}\left(\zeta_{3}\right)\right]+T_{x}\left(\zeta_{3}\right)\right)\right]\\ v^{\prime \prime } & =v^{\prime }⊙\exp\left[\frac{\epsilon }{2}S_{v}\left(\zeta_{4}\right)\right]-\frac{\epsilon }{2}\left(\partial_{x}U\left(x^{\prime \prime }\right)⊙\exp\left[\epsilon Q_{v}\left(\zeta_{4}\right)\right]+T_{v}\left(\zeta_{4}\right)\right). \end{array}$$

Here, $\zeta =\left(x,\partial_{x}U\left(x\right),t\right)$ represents the full state and excludes *v*. The three newly introduced functions *T*, *Q* and *S* are all neural networks. Scale items exp(S(ζ)) and exp(Q(ζ)) utilize gradient information of the target distribution as input, which will take up much computational budget. $m^{t}$ is a fixed random binary mask that determines which variables are updated. The update scheme first updates a subset of the coordinates of *x* [13], and then updates the rest subset to make transition kernels more expressive.

However, L2HMC has poor performance in far-distance multi-modal distributions. When using a small batch size, it will be hard to converge to the target distribution or traverse between correct modes in our empirical experiments, which indicates that L2HMC can not effectively and correctly use the gradient information of the target distribution to help build high-performance transition kernels.

Since the large step size will introduce more errors when traversing along iso-probability contours, there is a trade-off between a high acceptance rate and high diversity of samples. For the training objective (Equation (17)) of L2HMC, the two aspects are all considered, but they do not have a relationship of adversarial form here. Moreover, the order of magnitude of $\delta \left(\xi ,\xi^{★}\right)$ and $A\left(\xi^{★}|\xi \right)$ seems to be different. $A\left(\xi^{★}|\xi \right)$ represents the acceptance rate which is no more than 1, while $\delta \left(\xi ,\xi^{★}\right)$ represents the Euclidean distance between samples that can be much greater than 1. When we optimizer the parameters through gradient descent with the objective of larger $\delta \left(\xi ,\xi^{★}\right)A\left(\xi^{★}|\xi \right)$, the influence of the two items on the gradient are obviously different. In other words, the trained sampler may have a high acceptance rate but high autocorrelation or vice versa under the loss function, which will bring bad empirical results. We will discuss the situation in detail in Section 5.

## 5. Experiment

In this section, we evaluate the performance of NVP-MC. We compare our method with A-NICE-MC(NICE), L2HMC and vanilla RNVP on four challenging distributions using the effective sample size (ESS) [35]. For vanilla RNVP, we firstly sample some correct sample points of the target distribution, then directly train the RNVP flows to capture the target distribution. Since the method is firstly inspired by Normalizing Flow(NF) [37], we use NF to represent the baseline method in our experiments. Then we compare with the three methods by evaluating maximum mean discrepancy (MMD) [38]. Similar to experiments in [13], we build a challenging mixture of Gaussians to show the shortcomings of parameterized gradient-based transition kernels. We demonstrate the sample density plots generated by the four different methods. Finally, we conduct experiments on nine real datasets using Bayesian logistic regression [39] to showcase the practicability of the proposed method.

### 5.1. Performance Indexes

ESS can reflect the magnitude of the autocorrelation or the number of “effective samples” of the samples, which is defined as:

(19) $$\mathrm{ESS}=N/\left[1+2\times \sum_{s=1}^{\infty }\rho \left(s\right)\right],$$

where *N* represents the total sample number. We use the same computation method as [15] to estimate $\rho_{s}$:

(20) $${\hat{\rho }}_{s}=\frac{1}{{\hat{\sigma }}^{2}\left(N-s\right)}\sum_{n=s+1}^{N}\left(x_{n}-\hat{\mu }\right)\left(x_{n-s}-\hat{\mu }\right),$$

where $\hat{\mu }$ and $\hat{\sigma }$ are the empirical mean and variance obtained from the independent sampler. Similar to [40], when the autocorrelation goes below 0.05 we truncate the sum to reduce the noise of large lags.

*MMD* can measure the difference between samples drawn from two distributions *X* and *Y*, which takes the form:

(21) $$\begin{array}{cc} MMD^{2}\left[X,Y\right]= & \frac{1}{M^{2}}\sum_{i,j=1}^{M}\kappa \left(x_{i},\;x_{j}\right)-\frac{2}{MN}\sum_{i,j=1}^{M,N}\kappa \left(x_{i},\;y_{j}\right)\\ & +\frac{1}{N^{2}}\sum_{i,j=1}^{N}\kappa \left(y_{i},\;y_{j}\right), \end{array}$$

where *M* represents the sample number in *X* while *N* represents the sample number in *Y*, and $\kappa \left(\cdot ,\cdot \right)$ is the kernel function which takes the form:

(22) $$\kappa \left(x,y\right)={\left(1.0+x^{⊤}y\right)}^{2}.$$

Through evaluating *MMD*, we can justify the convergence of the proposed method. The lower the *MMD* value is, the more rapid convergence the sampler has. We run the same computation procedure of *MMD* for 10 times and record the mean and variance as our final results.

### 5.2. Varieties of Challenging Energy Functions

**Figure 3:**
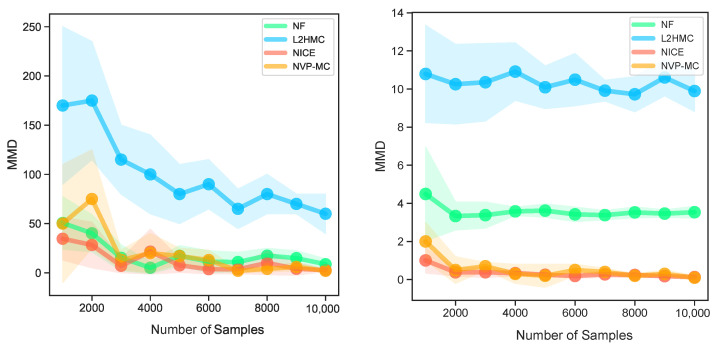
The performance of NF, L2HMC, NICE, NVP-MC on SCG (**left**) and MoG6 (**right**) distributions. In limited training steps, NF and L2HMC can not sample correctly from the target distributions compared with A-NICE-MC and NVP-MC.

We present an empirical evaluation for our trained sampler on varieties of synthetic 2D energy functions. For all energy functions, the largest ESS for all dimensions is shown in Table 1 and the MMD value is demonstrated in Figure 3. Then we further utilize the MoG experiment, to show the powerful exploration efficiency of the proposed method, compared with L2HMC.

For synthetic 2D target distributions we choose: Mixture of six Gaussians (MoG6): The analytic form of p(x) for MoG6 is:

$$p\left(x\right)=\frac{1}{6}\sum_{i=1}^{6}N\left(x|\mu_{i},\sigma_{i}\right),$$

where $\mu_{i}=\left[\sin\frac{i\pi }{3},\cos\frac{i\pi }{3}\right]$ and $\sigma_{i}=\left[0.5,\;0.5\right]$.

Gaussian funnel (GF): The energy function of the 2D funnel is

$$U\left(x\right)=\frac{1}{2}\left[{\left(\frac{x_{1}}{\sigma }\right)}^{2}+\frac{x_{2}^{2}}{\exp\left(x_{1}\right)}+\ln\left[2\pi \cdot \exp\left(x_{1}\right)\right]\right]$$

and we set σ=1.0.

Strongly correlated Gaussian (SCG): We rotate a diagonal Gaussian with variance $\left[10^{2},10^{-2}\right]$ by $\frac{\pi }{4}$, which takes the form:

$$p\left(x\right)=N\left(0,B\Sigma B^{T}\right),\;\Sigma =\left[\begin{array}{cc} 10^{-2} & 0\\ 0 & 10^{2} \end{array}\right],\;B=\left[\begin{array}{cc} 1/\sqrt{2} & -1/\sqrt{2}\\ 1/\sqrt{2} & 1/\sqrt{2} \end{array}\right].$$

The case is an extreme version of the example in [35].

Mixture of five rings (Ring5): The analytic form of the energy function of the mixture of 5 ring shaped target distributions is:

$$U\left(x\right)=\min\left(u_{1},\;u_{2},\;u_{3},\;u_{4},\;u_{5}\right),\;$$

where $u_{i}={\left(\sqrt{x_{1}^{2}+x_{2}^{2}}-i\right)}^{2}/0.04$.

Mixture of Gaussians (MoG2): This is a mixture of two isotropic Gaussians separated by the distance of $10\sqrt{2}$:

$$p\left(x\right)=0.5*N\left(x|\mu_{1},\sigma_{1}\right)+0.5*N\left(x|\mu_{2},\sigma_{2}\right),$$

where $\mu_{1}=\left[5,5\right]$, $\mu_{2}=\left[-5,-5\right]$ and $\sigma_{1}^{2}=3,\sigma_{2}^{2}=0.05$. The MoG2 distribution is more challenging than the example used to show that L2HMC has better mixing performance than A-NICE-MC in [13].

Ill-Conditioned Gaussian (ICG): This is a Gaussian distribution with diagonal covariance spaced log-linearly between 0.01 and 100, which has the same analytic expression with SCG.

We use the same hyperparameters for all density-based experiments. Specifically, we construct the transition kernels with three coupling layers to ensure that both *x* and *v* could get fully updated. We get initial samples through HMC without MH steps with 6 leapfrog steps and step size ϵ=0.3, and discard the first 1000 steps as burn-in steps. The remain samples are saved in a buffer, after each training epoch of transition kernels, the samples in the buffer will be discard by 0.5 and add the new accepted samples into the buffer. At each training epoch, the transition kernels will move 5000 steps. In each coupling layer, the *S* and *T* are 3 layers neural networks. The discriminator is also 3 layer neural networks with 400 units and activated with leaky rectified linear units. We train our model by Adam [41] optimizer with batch size of 32 and $beta_{1}=0.5$, $beta_{2}=0.9$ for $D_{loss}$ and $G_{loss}$. The learning rate is set to be 0.0005 for the $D_{loss}$, and 0.0003 for the $G_{loss}$. To make the training process more stable, we clip the gradient of all the neural networks by −8 and 8.

As shown in Figure 3, A-NICE-MC and NVP-MC have lower MMD among the two distributions. When we use less than 4000 samples to compute MMD, the MMDs variance of NVP-MC is larger than A-NICE-MC. Its because that NVP-MC explores the target distributions in a more extensive way, which brings more noise, however, the exploration can bring NVP-MC samples with higher quality. When we use more than 4000 samples to compute MMD, the variance of MMD of NVP-MC is almost the same as A-NICE-MC, while the two methods all achieve the lowest MMD compared with NF and L2HMC.

**Figure 4:**
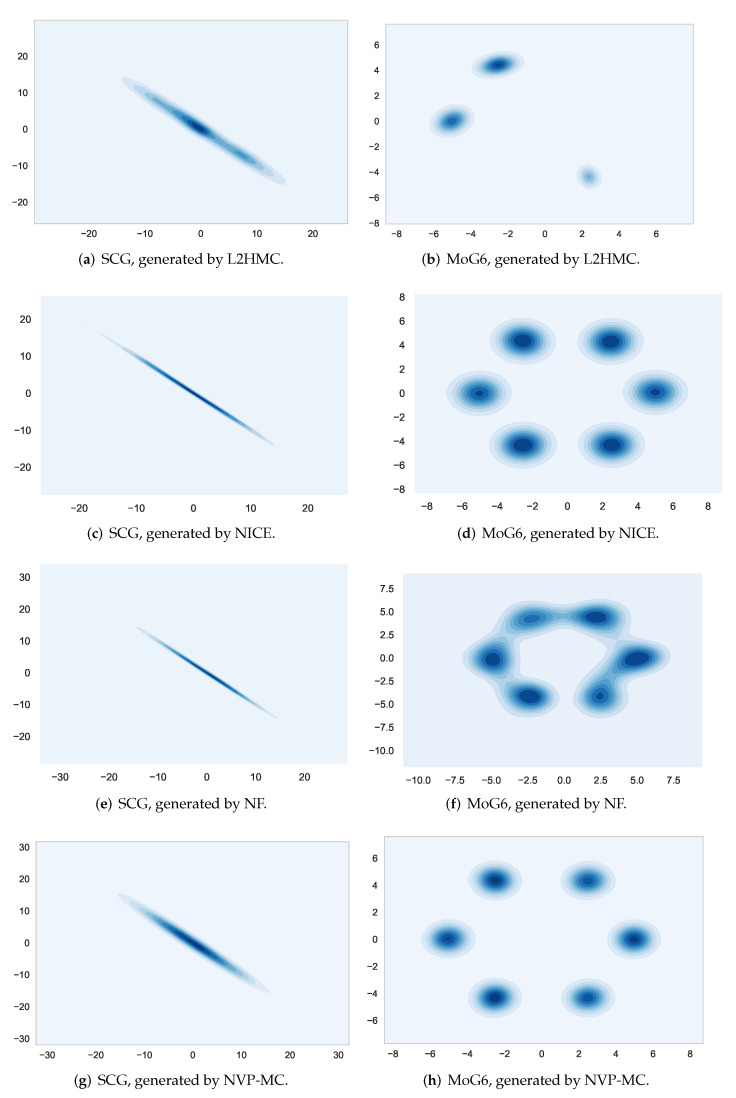
Density plots of samples from L2HMC, A-NICE-MC, NF and NVP-MC.

We further demonstrate the sample density plots generated by the four different methods. As Figure 4 shows, L2HMC and NF cannot find the target distributions of SCG and MoG6, while NVP-MC still performs well, for NVP-MC exploits HMC without MH steps as the exploration operator to get higher mixing performance. Although NICE is able to find the target distributions of SCG and MoG6, it has a large error (lower ESS) while NVP-MC is able to sample from the target distribution precisely.

To estimate the quality of samples, we train NVP-MC, A-NICE-MC and L2HMC for 10,000 iterations with the same batch size and record the minimum value of ESS for all dimensions. Because we choose the same model size as A-NICE-MC (neural networks with 3 hidden layers with 400 (1024 for the 50-*d* ICG), the training time is similar. Therefore, there is no need for us to construct experiments to evaluate ESS per second (ESS/S) which is mentioned in [15]. L2HMC consumes far more time than A-NICE-MC and NVP-MC but gets the ESS value that does not match the effort. As Table 1 illustrates, NVP-MC can get significant improvement in ESS in all distributions compared with A-NICE-MC.

**Figure 5:**
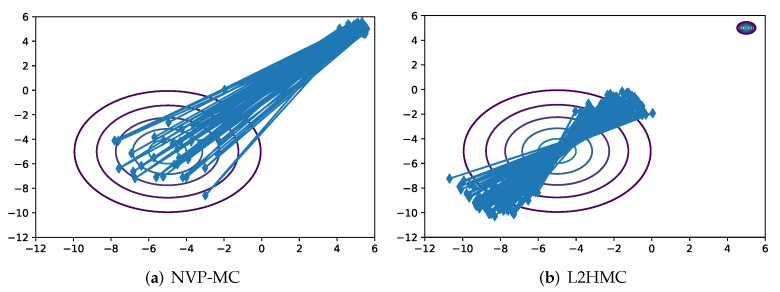
NVP-MC (**a**) can correctly and faster mix between modes in limited training steps and batch sizes compared with L2HMC (**b**).

**Figure 6:**
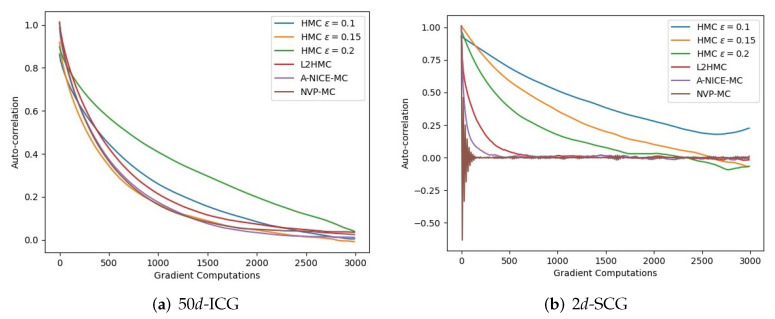
Autocorrelation with respect to gradient evaluation steps for 50-*d* ICG and 2*d*-SCG.

To further validate the performance of the proposed method in far-distance multi-modal distributions, we build on the MoG experiment presented in [13]. We increase the distance between modes by $\sqrt{2}$ and use the same variance. We run L2HMC and NVP-MC 10,000 iterations respectively, and use the same batch size for the two algorithms. As shown in Figure 5, although L2HMC introduces the gradient information of the target distribution, it can not correctly sample from the target distribution while NVP-MC can mix quickly between the modes, which indicates that it may be inappropriate to introduce gradient information directly into parameterized transition kernels. Gradient information is informative in the exploration stage, but not the training stage. By dividing the training process into the two stages, we can remove the restriction of exact sampling in the exploration stage and the proposed method can have powerful exploration ability. In the training stage, adversarially training can learn the geometry of the target distribution by minimizing the loss function. The exact samples can be obtained through accepting the samples generated from the transition kernels by using MH steps. Moreover, L2HMC needs twice times than NVP-MC to train the transition kernels in the training stage, and a trained NVP-MC can sample from the target distribution six times faster than HMC. We also report the autocorrelation of NVP-MC and the compared methods. As seen in Figure 6, the samples collected by NVP-MC have the fastest drop in autocorrelation with respect to gradient evaluations in target distribution of 2*d*-SCG. In high-dimensional target distribution of 50*d*-ICG, the rate of autocorrelation decline of NVP-MC is similar to that of NICE-MC and slightly faster than that of L2HMC.

### 5.3. Bayesian Logistic Regression

In this section, we conduct the experiments on nine real datasets using Bayesian logistic regression. To show the practicability of the proposed method, we sample from the posterior distribution and compare NVP-MC with HMC, logistic regression (LR) [42] and variational Bayesian logistic regression (VBLR), which are all the widely applicable methods in Bayesian logistic regression [43]. Given a conditional distribution p(Y|X) parameterized by the logistic distribution, the goal of LR is to maximize the likelihood function and get the optimized parameters to predict the class of the data. We consider nine datasets from UCI repository [44]: Pima (Pi), Haberman (HA), Blood (BL), Immunotherapy (IM), Indian (IN), Mammographic (MA), Heart(HE), German (GE) and Australian (AU) and evaluate the accuracy rate and area under the receiver operating characteristic curve (AUC) [45]. To improve the stability of the models, we normalize all datasets to have zero mean and unit variance. We set the normal distribution N(0, I) as the prior distribution of parameters, and use the same data partition for all experiments.

We set the dimension of the auxiliary variable *v* to 35 for every real dataset experiment. At each training epoch, the transition kernels will move 5000 steps. In each coupling layer, the *S* and *T* are 3 layers neural networks with 400 units for input and output layer, 800 units for the hidden layer, and each layer is not activated. The discriminator is 3 layer neural networks with 800 units and activated with leaky rectified linear units. As for the optimizer, we use the same setting as in the synthetic 2D target distribution experiments.

As Table 2 and Table 3 illustrated, NVP-MC can obtain better performance in almost all datasets, which indicates that the proposed method can sample from the posterior distribution more accurately.

## 6. Conclusions

In this study, we develop the adversarially training MC, non-volume-preserving transition kernels, and exploit a novel scheme to train MCMC kernels with promising mixing performance. Compared with existing gradient-based sample methods, the proposed method can leverage gradient information more efficiently, and explore the target distribution faster. The experiments in various challenging distributions and real datasets show that the proposed method outperforms other state-of-the-art MCMC methods and is promising for practical uses.

## Figures and Tables

**Table 1:** The ESS of NVP-MC, A-NICE-MC, L2HMC, and HMC. Data in bold represent best results.

Target	NVP-MC	A-NICE-MC	L2HMC	HMC
MoG6	568.2	320.0	311.2	1.0
GF	834.3	270.0	304.1	8.0
SCG	1000.0	539.4	497.0	0.48
Ring5	200.3	155.6	69.1	0.43
50-*d* ICG	0.83	0.29	0.78	0.02

**Table 2:** Classification accuracy for LR, VBLR, HMC and NVP-MC. Data in bold represent best results.

Dataset	LR	VBLR	HMC	NVP-MC
HA	69.3 ± 0.2	69.3 ± 0.1	69.3 ± 0.2	69.4 ± 0.1
PI	76.6 ± 0.2	76.2 ± 0.1	76.6 ± 0.1	76.8 ± 0.1
MA	82.5 ± 0.3	83.1 ± 0.1	83.1 ± 0.1	83.1 ± 0.1
BL	76.0 ± 0.2	76.0 ± 0.2	76.0 ± 0.3	76.1 ± 0.2
IM	77.7 ± 0.3	77.8 ± 0.4	83.2 ± 0.2	83.3 ± 0.4
IN	75.8 ± 0.3	73.2 ± 0.2	73.2 ± 0.2	73.8 ± 0.2
HE	75.9 ± 0.2	75.9 ± 0.2	75.9 ± 0.2	76.1 ± 0.2
GE	71.5 ± 0.1	71.5 ± 0.1	72.5 ± 0.2	73.2 ± 0.1
AU	86.9 ± 0.2	87.6 ± 0.2	87.6 ± 0.2	87.7 ± 0.2

**Table 3:** AUC for LR, VBLR, HMC and NVP-MC. Data in bold represent best results.

Dataset	LR	VBLR	HMC	NVP-MC
HA	62.7 ± 0.1	63.2 ± 0.1	63.0 ± 0.2	63.3 ± 0.1
PI	79.2 ± 0.2	79.3 ± 0.1	79.3 ± 0.1	79.4 ± 0.1
MA	89.9 ± 0.1	89.8 ± 0.1	89.9 ± 0.1	89.9 ± 0.1
BL	73.5 ± 0.3	73.4 ± 0.3	74.4 ± 0.3	73.6 ± 0.2
IM	76.7 ± 0.3	78.5 ± 0.5	89.2 ± 0.2	89.3 ± 0.4
IN	73.2 ± 0.3	73.2 ± 0.2	72.4 ± 0.2	72.7 ± 0.2
HE	80.1 ± 0.2	81.3 ± 0.2	82.2 ± 0.3	81.9 ± 0.2
GE	74.7 ± 0.2	75.5 ± 0.2	76.7 ± 0.3	76.7 ± 0.1
AU	92.5 ± 0.2	93.9 ± 0.2	93.9 ± 0.3	93.9 ± 0.2

## Data Availability

Data available in a publicly accessible repository. The data presented in this study are openly available in UCI Machine Learning Repository at http://archive.ics.uci.edu/ml/index.php (accessed on 13 March 2022).
